# Spatial Memory Dysfunction Induced by Vitamin C Deficiency Is Associated with Changes in Monoaminergic Neurotransmitters and Aberrant Synapse Formation

**DOI:** 10.3390/antiox7070082

**Published:** 2018-06-29

**Authors:** Stine Normann Hansen, Anne Marie V. Schou-Pedersen, Jens Lykkesfeldt, Pernille Tveden-Nyborg

**Affiliations:** Section for Experimental Animal Models, Department of Veterinary and Animal Sciences, University of Copenhagen, Thorvaldensvej 57, Ground Floor, 1870 Frederiksberg C, Denmark; snoha@sund.ku.dk (S.N.H.); am.schoupedersen@sund.ku.dk (A.M.V.S.-P.); jopl@sund.ku.dk (J.L.)

**Keywords:** Cavia porcellus, memory deficit, hippocampus, synapse formation, monoaminergic neurotransmitters

## Abstract

Vitamin C (vitC) is important in the developing brain, acting both as an essential antioxidant and as co-factor in the synthesis and metabolism of monoaminergic neurotransmitters. In guinea pigs, vitC deficiency results in increased oxidative stress, reduced hippocampal volume and neuronal numbers, and deficits in spatial memory. This study investigated the effects of 8 weeks of either sufficient (923 mg vitC/kg feed) or deficient (100 mg vitC/kg feed) levels of dietary vitC on hippocampal monoaminergic neurotransmitters and markers of synapse formation in young guinea pigs with spatial memory deficits. Western blotting and high performance liquid chromatography (HPLC) were used to quantify the selected markers. VitC deficiency resulted in significantly reduced protein levels of synaptophysin (*p* = 0.016) and a decrease in 5-hydroxyindoleacetic acid/5-hydroxytryptamine ratio (*p* = 0.0093). Protein expression of the *N*-methyl-d-aspartate receptor subunit 1 and monoamine oxidase A were reduced, albeit not reaching statistical significance (*p* = 0.0898 and *p* = 0.067, respectively). Our findings suggest that vitC deficiency induced spatial memory deficits might be mediated by impairments in neurotransmission and synaptic development.

## 1. Introduction

Vitamin C (vitC) deficiency is a surprisingly common nutritional insufficiency affecting around 15% of the Western population [1,2,3], including subpopulations such as pregnant women and young children [4,5]. The vitamin is a powerful antioxidant, and crucial in the developing brain, where antioxidant defenses are still immature and a high cellular metabolism gives rise to increased levels of reactive oxygen species [6,7]. In the face of dietary depletion, vitC levels in the brain are maintained at approximately 25% of saturated values—as opposed to more extensive reductions in most other organs [8,9], suggesting that the nutrient is of high importance in this tissue. Early life vitC deficiency has been shown to cause impairments in spatial memory, decrease hippocampal volume and neuron numbers in guinea pigs [10,11], a species dependent on dietary vitC akin to humans [12,13]. However, the molecular mechanisms behind the recorded memory deficits are largely undisclosed. 

In addition to being pivotal in maintaining brain redox homeostasis, cerebral vitC is linked to glutamatergic neurotransmission and protection against glutamate induced neuronal excitotoxicity [14,15,16]. VitC is also involved in monoaminergic neurotransmission (dopamine, norepinephrine and possibly serotonin; 5-hydroxytryptamine (5-HT)) [16,17,18]. Though vitC’s specific role in the brain has not been conclusively elaborated, it acts as a co-factor donating electrons to dopamine-β-hydroxylase and is suggested to play a role in monoaminergic neurotransmitter synthesis by keeping the co-factor tetrahydrobiopterin (BH4) in its reduced form [14,16,18,19]. Furthermore, alterations of synaptic structure and associated proteins and receptors have been shown to underlie cognitive dysfunction, behavioral changes and memory formation [20,21,22]. Thus, either through secondary (antioxidant-mediated) or direct effects on neuronal signaling pathways and dendrite development, vitC deficiency can be speculated to lead to aberrant neurotransmission in the hippocampus, hereby causing the reported spatial memory deficits in young guinea pigs.

In addition, dendrite development is crucial in establishing and maintaining synaptic contacts between neurons [21,23], and decreased dendritic arborization and clustering of the excitatory α-amino-3-hydroxy-5-methyl-4-isoxazolepropionic acid (AMPA) subtype and glutamate receptor subunit (GluR)1 following ablation of neuronal vitC transport has been shown in vitro, suggesting vitC to be a key component for neuronal outgrowth and subsequent signal transduction [14]. We have previously shown impaired hippocampal function measured by decreased spatial memory performance in the Morris Water Maze, in coherence with significantly reduced hippocampal neuron numbers in guinea pigs subjected to vitC deficiency during early life and until reproductive maturity [10]. With off-set in these findings, the current study explores the hypothesis that the recorded memory deficits resulting from vitC deficiency are caused by decreased neuronal signal transmission by reducing monoaminergic neurotransmitters and/or synapse formation and function.

## 2. Materials and Methods 

### 2.1. Animals

All experiments were approved by the Danish Animal Experiments Inspectorate under the Ministry of Environment and Food (2007/561-1298). The behavioral and histological findings from the in vivo study have previously been published [10]. 

Briefly, 27, five to six days old female Dunkin Hartley guinea pigs (Charles River Laboratories, Kisslegg, Germany) were weight-stratified and randomly allocated to receive either 923 mg/kg vitC diet by analysis (CTRL, *n* = 15) or 100 mg/kg vitC diet by analysis (DEF, *n* = 12) (Special Diets Services, Dietex International Ltd., Witham, UK) [10]. All animals were inspected and handled daily by trained personnel and allowed ad libitum access to feed, hay (without vitC by analysis) and water. At 52–53 days of age, the animals were subjected to the Morris Water Maze (MWM) test regime, as previously published [10]. At 60–61 days of age, the animals were anesthetized with 0.175 mL/100 g bodyweight Zoletil mix, consisting of 0.465 mg/mL Zoletil-50 (Virbac SA, Carros Cedex, France), 2 mg/mL Xylazin (Narcoxyl, Intervet Int., Boxmeer, The Netherlands), and 1 mg/mL butorphanol (Torbugesic, ScanVet, Fredensborg, Denmark) and briefly supplemented with carbon dioxide inhalation. After disappearance of voluntary reflexes, an intracardiac blood sample was obtained and the animal euthanized by exsanguination [10]. 

The brain was excised, washed in ice-cold phosphate buffered saline (PBS), and divided into hemispheres. A subset of the hemispheres was randomly allocated to stereological analyses (previously published) [10]. The hippocampus was removed from the remaining left or right hemisphere as determined by randomization, snap frozen in liquid nitrogen and stored at −80 °C until further processing. 

### 2.2. Monoaminergic Neurotransmitters

The analysis of monoaminergic neurotransmitters in the hippocampus was carried out by high performance liquid chromatography (HPLC) as previously described [24]. All samples were analyzed in triplicate and in a randomized order.

### 2.3. Protein Extraction

The protein was extracted as previously described with some modifications [25]. In short, 40 mg of frozen hippocampal tissue was excised on ice before adding 500 μL RIPA buffer (50 mmol/L tris pH 8.0, 150 mmol/L sodium chloride, 1% Triton X-100, 0.5% sodium deoxycholate and 0.1% sodium dodecyl sulfate) with 1:100 protease inhibitor cocktail (Sigma-Aldrich, Darmstadt, Germany) and 1:100 phosphatase inhibitor cocktail (Sigma-Aldrich, Darmstadt, Germany) and homogenized by mortar and pestle on ice. The samples were centrifuged for 10 min at 12,000 rpm at 4 °C and the supernatant divided in aliquots and stored at −80 °C. In addition, another 10 mg of hippocampal tissue was excised on ice before adding 250 μL of Tissue Protein Extraction Reagent (T-PER) (Thermo Fisher Scientific, Waltham, MA, USA) with 1:100 protease inhibitor cocktail (Sigma-Aldrich, Darmstadt, Germany) and 1:100 phosphatase inhibitor cocktail (Sigma-Aldrich, Darmstadt, Germany). The samples were centrifuged at 10,000× *g* for five minutes at 4 °C according to manufacturer’s instructions and the supernatant divided in aliquots and stored at −80 °C. Two animals from the CTRL group were excluded for technical reasons, leaving the CTRL group size at *n* = 13. Protein concentrations were determined using a commercial BCA kit according to manufacturer’s instructions (Merck Millipore, Darmstadt, Germany).

### 2.4. Western Blotting

The Western blotting procedure was carried out on samples in duplicates and in a randomized order, as previously described [25]. The amount of protein (determined by a dilution series) was adjusted to 11.25 μL with ultrapure water before adding 3.75 μL Laemmli buffer (Hercules, CA, USA) with 1:10 mercaptoethanol (Sigma-Aldrich, Darmstadt, Germany). After denaturing for 10 min at 70 °C, the samples were loaded on a 7.5% Criterion™ TGX™ Precast Midi Protein Gel, 26 well (Bio Rad, Hercules, CA, USA, 15 μL/well) and the electrophoresis was run for approximately 40 min before transferring proteins to a polyvinylidene diflouride (PVDF) membrane. Samples were normalized to total protein levels (REVERT™ Total Protein Stain, Li-Cor, Lincoln, NE, USA). Every blot included positive and negative control samples and a calibrator to account for inter-membrane variation.

The following antibodies were applied: Anti-monoamine oxidase A (MAOA) (ab126751, Abcam, Cambridge, UK, 1:2000, 10 μg protein) with IRDye^®^ 680RD Donkey-anti-Rabbit IgG (Li-Cor, Lincoln, NE, USA; 1:15,000) as the secondary antibody, anti-GluR1 (ab183797, Abcam, Cambridge, UK, 1:1000, 30 μg protein) with IRDye^®^ 680RD Donkey-anti-Rabbit IgG (Li-Cor, Lincoln, NE, USA; 1:15,000) as the secondary antibody, anti-tyrosine hydroxylase (TH) (ab75875, Abcam, Cambridge, UK, 1:500, 40 μg protein) with IRDye^®^ 680RD Donkey-anti-Rabbit IgG (Li-Cor, Lincoln, NE, USA; 1:15,000) as the secondary antibody, anti-tryptophan hydroxylase (TpH) 2 (AV34141, Sigma-Aldrich, Darmstadt, Germany, 1:2000, 20 μg protein) with IRDye^®^ 680RD Donkey-anti-Rabbit IgG (Li-Cor, Lincoln, NE, USA; 1:15,000) as the secondary antibody, anti-post-synaptic-density-protein-95 (PSD-95) (D27E11 3450, Cell Signaling Technology, Boston, MA, USA, 1:1000, 20 μg protein) with IRDye^®^ 680RD Donkey-anti-Rabbit IgG (Li-Cor, Lincoln, NE, USA; 1:15,000) as the secondary antibody, anti-synaptophysin (ab8049, Abcam, Cambridge, UK, 1:1000, 20 μg protein) with IRDye^®^ 680RD Donkey-anti-Mouse IgG (Li-Cor, Lincoln, NE, USA; 1:15,000) as the secondary antibody, anti-neuronal nuclei marker (NeuN) (MAB377, Merck Milipore, Burlington, MA, USA, 1:2000, 20 μg protein) with IRDye^®^ 680RD Donkey-anti-Mouse IgG (Li-Cor, Lincoln, NE, USA; 1:15,000) as the secondary antibdy, anti-glial fibrillary acidic protein (GFAP) (ab7260, Abcam, Cambridge, UK, 1:20,000, 20 μg protein) with IRDye^®^ 800CW Donkey-anti-Rabbit IgG (Li-Cor, Lincoln, NE, USA; 1:15,000) as the secondary antibody and anti-*N*-methyl-d-aspartate receptor subunit 1 (NMDAR1) (ab77264, Abcam, Cambridge, UK, 1:2000, 20 μg protein) with IRDye^®^ 800CW Donkey-anti-Goat IgG (Li-Cor, Lincoln, NE, USA; 1:15,000) as the secondary antibody. All secondary antibodies were applied for one hour at r/t.

Synaptophysin and NMDAR1 were analyzed using T-PER Tissue Protein Extraction Reagent (Thermo Fisher Scientific, Waltham, MA, USA) extracted protein and the remaining markers using RIPA extracted protein. The subsequent analyses of staining intensity were completed through Image Studio 5.2 (Li-Cor, Lincoln, NE, USA) by an observer blinded to the experimental groups.

### 2.5. Statistics

Statistical analyses were performed by GraphPad Prism 7 (GraphPad Software, La Jolla, CA, USA). Student’s *t*-test was used for both the neurotransmitter and Western blot analyses. In the event of nonhomogeneous variances, the data was log-transformed or Welch’s *t*-test applied. All results are presented as mean ± SD (standard deviation) or geometric mean (95% confidence interval). A *p*-value < 0.05 was considered statistically significant.

## 3. Results

The current study is an extension of a previously published in vivo study [10]. In brief, the dietary regimes resulted in ascorbate (Asc; the reduced and active form of vitC) plasma concentrations of 104 ± 34.2 µM in CTRL and 8.5 ± 3.7 µM in DEF, and brain Asc levels of 1256 ± 87.4 nmol/g tissue and 519 ± 99.6 nmol/gram tissue in CTRL and DEF, respectively. Subjected to the Morris Water Maze at day 52–53 of age (around the onset of reproductive maturity), DEF animals displayed significantly impaired performance in the retention test; reduced time spent in platform quadrant, reduced number of crossings of platform area and increased time to first platform area hit (*p* < 0.05, *p* < 0.01 and *p* < 0.05 respectively), compared to CTRL counterparts. A significantly reduced ability to apply a spatial swim pattern was seen in DEF animals compared to CTRL. Stereological quantification of the hippocampus revealed significantly reduced neuron numbers in DEF in all investigated areas (the dentate gyrus, cornu amonis 1 and 2 + 3) [10].

To detect differences in hippocampal neuronal signaling, monoaminergic neurotransmitters and selected metabolites were investigated. The results are shown in Table 1. There was a decreased 5-hydroxyindoleacetic acid (5-HIAA)/5-HT ratio in the DEF group compared with the CTRL (*p* = 0.0093). No other neurotransmitters or their metabolites displayed any differences between the two groups. Dopamine and dopamine metabolites were found to be below detection limit (12 nM for homovanilic acid (HVA), 3.6 nM for 3,4-dihydroxyphenylacetic acid (DOPAC) and 10 nM for dopamine).

**Figure 1:**
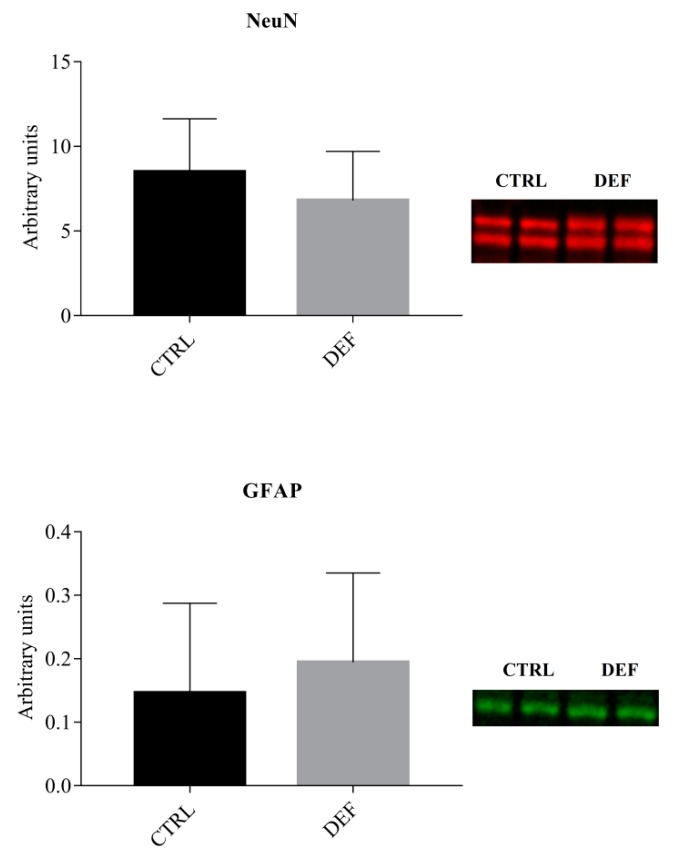
Cellular markers in the hippocampus. The figure depicts the Western blot analyses of the levels of the investigated cellular markers in the hippocampus relative to total protein levels. The two bands in the NeuN samples are consistent with splice variants (confirmed by the manufacturer). No differences between the two groups were detected by Student’s *t*-test. Data is shown as mean ± SD, SD: standard deviation. GFAP: Glial-fibrillary-acidic-protein, NeuN: Neuronal nuclei marker. CTRL: Control animals (*n* = 13), DEF: Deficient animals (*n* = 12).

**Figure 2:**
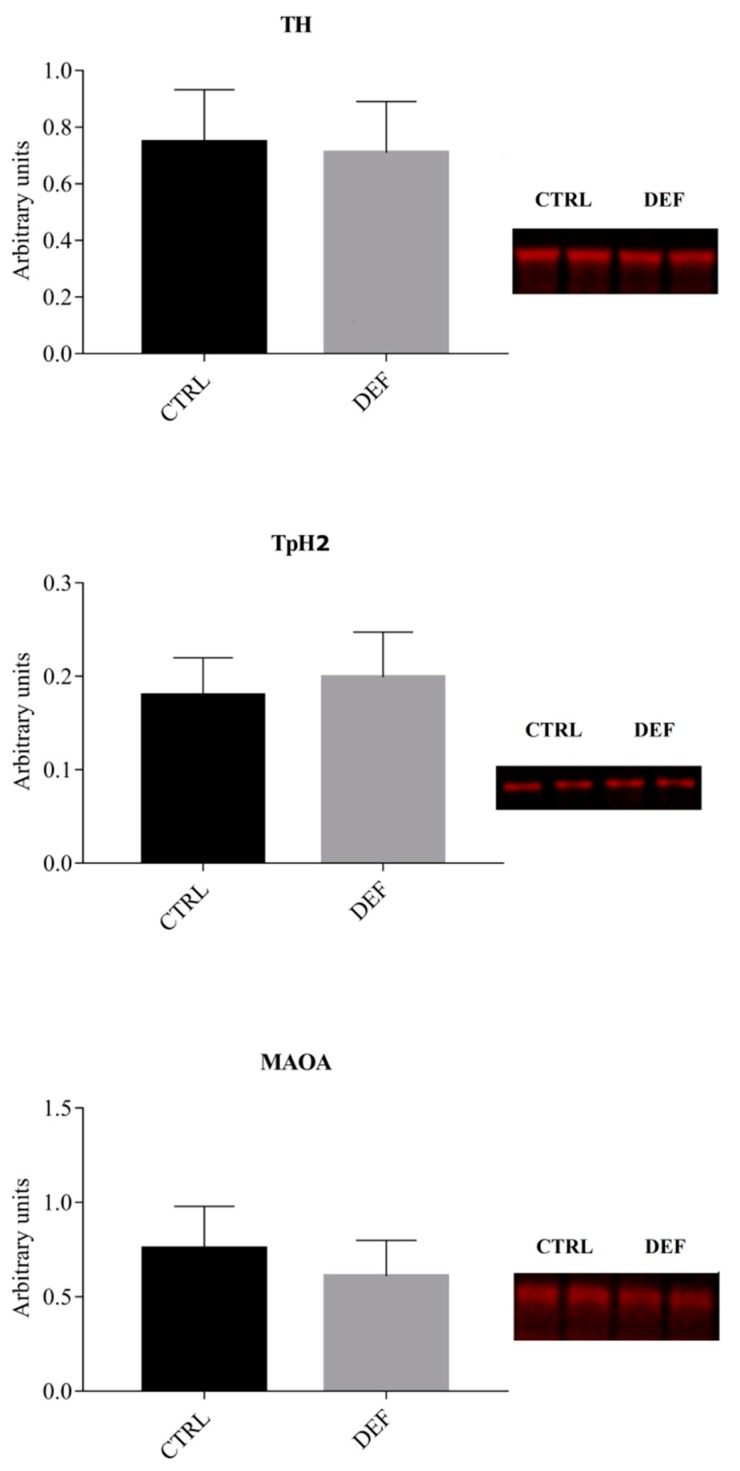
Monoamine synthesizing proteins in the hippocampus. The figure shows the levels of markers associated with monoaminergic neurotransmission in the hippocampus as detected by Western blotting. MAOA may be approaching a decrease in DEF (*p* = 0.0844), albeit not reaching significance. The expression of the other investigated markers was not different between groups. Data is displayed as mean ± SD and analyzed by Student’s *t*-test. TH: Tyrosine hydroxylase; TpH2: Tryptophan hydroxylase 2; MAOA: Monoamine-oxidase A; CTRL: Control animals (*n* = 13); DEF: Deficient animals (*n* = 12).

**Figure 3:**
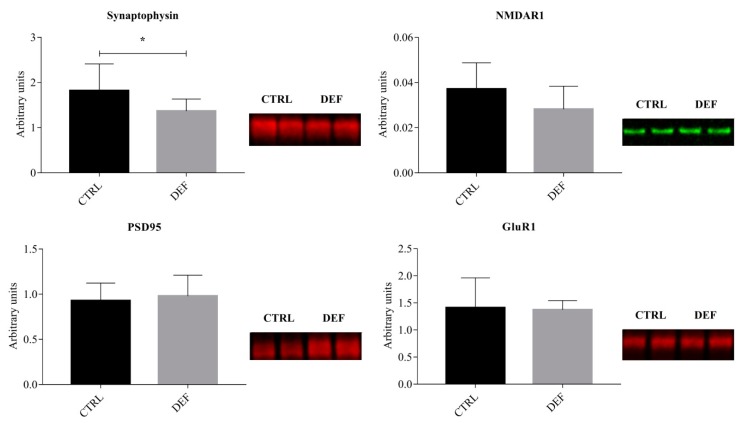
Synapse markers in the hippocampus. The figure shows the results from the Western blotting of markers of synapse formation. Synaptophysin is down-regulated in the DEF group (*p* = 0.0160), while *N*-methyl-d-aspartate receptor 1 (NMDAR1) displays a tendency for down-regulation (*p* = 0.0525). Data is displayed as mean ± SD and analyzed by Student’s or Welch’s *t*-test. *: *p* < 0.05; PSD-95: Post-synaptic-density-protein-95; CTRL: Control animals (*n* = 13); DEF: Deficient animals (*n* = 12); GluR1: α-amino-3-hydroxy-5-methyl-4-isoxazolepropionic acid receptor subunit 1.

To assess whether the imposed state of vitC deficiency in the brain would result in alteration of hippocampal protein expression, Western blot analyses were performed on selected markers, including markers of neuronal maturation (NeuN) and astrocytes (GFAP), as well as more specific markers linked to synapse formation and neurotransmission, such as synaptophysin and NMDAR1. The results from the Western blot analyses are shown in Figure 1, Figure 2 and Figure 3.

Though the depicted expression patterns may indicate reduced NeuN levels and increased GFAP levels in DEF animals, this could not be confirmed statistically. Hence no differences were observed in overall markers of neuronal and glial cells, NeuN and GFAP, respectively, between the two diet groups (Figure 1). 

To determine the effects of vitC deficiency on the rate limiting enzymes involved in the metabolism (synthesis and removal) of monoaminergic neurotransmitters, the expression of tyrosine hydroxylase (TH; linked to dopamine and norepinephrine synthesis), tryptophane hydroxylase 2 (TpH2; linked to serotonin synthesis) and monoamine oxidase (MAOA; linked to the removal of dopamine, norepinephrine and serotonin) was assessed. No statistically significant difference between groups could be detected for TH and TpH2. MAOA expression did not reach statistical significance between groups (*p* = 0.0844) (Figure 2). 

To evaluate an effect of vitC deficiency on markers of synaptic function (both pre- and postsynaptic), the expression of synaptophysin, NMDAR1, PSD-95 and GluR1 was investigated by Western blotting. A significant decrease in synaptophysin in the DEF group was evident (*p* = 0.0160), while NMDAR1 approached a decrease in DEF (*p* = 0.0525) (Figure 3), albeit not reaching statistical significance. 

Expression patterns suggest a NeuN decrease and GFAP increase in DEF, although not substantial enough to reach a significant difference between groups. To assess if a potential difference in neuronal expression was reflected in the expression of additional markers adhering to neuronal function, the expression of synaptic markers NMDAR1, GluR1, synaptophysin and post-synaptic-density-protein-95 relative to neuronal marker NeuN was calculated (relative expression = marker/NeuN). Likewise, the GFAP/NeuN expression ratio was compared to assess glia versus neuron ratio between groups. No statistically significant differences between groups were detected.

## 4. Discussion

The findings in this study suggest that a vitC deficiency-imposed impairment in spatial memory may be mediated by alterations in monoaminergic neurotransmitter metabolism and aberrant synapse formation in the hippocampus [10].

The decreased 5-HIAA/5-HT ratio in DEF animals supports a putative role of vitC in the metabolism of monoamine neurotransmitters, and that this may subsequently be affected by a state of deficiency, in this case likely due to a decreased 5-HT metabolism. Investigations in vitC deficient mice (*gulo−/−*) found regional alterations (striatum vs. cortex; hippocampus was not analyzed) in the serotonergic system in the brain [17], supporting that there are effects of vitC deficiency, but that these may be presented differently within brain areas.

No apparent difference in hippocampal TpH2 expression—the rate limiting enzyme in 5-HT synthesis [26]—was found between CTRL and DEF groups, in coherence with reports from mice embryos subjected to vitC depletion (SVCT2*−/−*) or moderate deficiency (SVCT2+/−) [18]. However it may be speculated that the decrease in 5-HT metabolism reflects a compensatory mechanism to keep 5-HT levels intact in response to a low 5-HT synthesis. Indeed, TpH2 is primarily expressed in the raphe nuclei in the brainstem [27,28] from where serotonergic neurons project to the hippocampus [29], hereby rendering TpH2 changes to be undetectable in the hippocampus. Serotonergic neurotransmission in the hippocampus is coupled to several functions including spatial memory [30,31]. An involvement of vitC in serotonergic neurotransmission, and subsequent deviations due to deficiency alongside decreased hippocampal neuron numbers could be a likely cause of the observed spatial memory deficits [10]. Whether additional changes in serotonergic neurotransmission are present—for example, reductions in the 5-HT1a and 5-HT4 receptors [31,32]—remains to be investigated.

Cortical levels of TH protein have been reported to be decreased in Asc depleted mice embryos (SVCT2*−/−*), but not significantly altered in moderately deficient mice embryos (SVCT2+/−) [18]. This is in agreement with the current finding of no effect on hippocampal TH levels during moderate vitC deficiency, supporting that significant reductions in TH requires extremely low levels of vitC.

The DEF animals displayed a decrease in synapse formation measured as reduced synaptophysin expression, which was positively correlated with Asc levels in the brain, emphasizing a potential direct effect of vitC deficiency on these markers. Synaptophysin is an abundant pre-synaptic vesicle protein involved in regulation of neurotransmitter exo- and endocytosis [33,34] and activity-dependent synapse formation [35]. The synaptophysin knockout mouse model, *Syn−/−*, display decreased learning and memory functions in the object novelty recognition test and MWM [36]. Additionally, several animal models of pathological brain development and memory deficits display decreased levels of synaptophysin [37,38,39], connecting this marker to the establishment of functional neuronal circuits. DEF animals of the current study displayed significant impairments in the MWM retention test, corresponding well with this finding.

A link between tyrosine phosphorylation of synaptophysin and long term potentiation (LTP)—the cellular hallmark of learning and memory—has been proposed, linking synaptophysin with glutamatergic neurotransmission [36,40,41]. LTP requires the activation of post-synaptic NMDA receptors (though there are also NMDA-independent forms of LTP) and is essential for spatial memory [42,43,44]. Though not reaching statistical significance, NMDAR1 expression may be decreased by reduced brain Asc status, and reflecting that vitC is important in NMDA receptor-mediated neurotransmission [16,45]. NMDA receptor activation by glutamate gives rise to downstream signaling cascades, increasing AMPA receptor insertion at the post-synaptic site [43]. In the current study, a decrease in the AMPA receptor subunit GluR1 was not detected in DEF, however, in vitro cultures of mouse neurons, devoid of the sodium-dependent vitC transporter 2, display reduced GluR1 clustering [14]. The absence of an effect on the GluR1 subunit in the present study could be due to the moderate state of vitC deficiency and/or represent compensatory mechanisms in vivo. Phosphorylation of GluR1 has been found to be imperative for LTP and memory retention, but not learning, in the MWM [46], suggesting that a decrease in GluR1 phosphorylation may govern the spatial cognitive deficits seen in the vitC deficient animals. Whether this is the case, or if other hippocampal AMPA receptor subunits, such as GluR2, known to be important for spatial memory [47], are affected, requires further investigation. 

The DEF animals in this study displayed decreased numbers of neurons in all areas of the hippocampus (the dentate gyrus and cornu ammonis 1 and cornu ammonis 2 and 3) [10]. Thus, a shift in the expression of synaptic markers relative to NeuN expression was explored to clarify any differences which could be masked by an underlying (though on its own un-significant) difference in NeuN expressing cells. The absence of differences in expression patterns relative to NeuN does not support such changes, nor do they support a putative presence of adaptive mechanisms to counteract, for example, decreases in neuronal number and/or a decrease in synapse formation. Furthermore, no alteration in the relative expression of GFAP to NeuN was found in deficient animals, showing that the cellular ratio between neurons and astrocytes was intact in vitC deficient animals. This finding supports that the cellular composition within the hippocampus was not affected by the imposed state of vitC deficiency, however, it is possible that the overall hippocampal volume was reduced in DEF animals hereby giving rise to the recorded reductions in neuronal number. A consistent reduction in overall hippocampal volume due to vitC deficiency in young guinea pigs has previously been reported [11]. Guinea pig specific antibodies of the investigated proteins are not currently commercially available and this represents a serious limitation of the recorded findings due to potential cross-species differences in antibody specificity. Although we included positive controls in all cases, the results of the present study should be verified once antibodies raised against guinea pig target proteins become available.

In conclusion, vitC deficiency-induced spatial memory deficits in young guinea pigs are mediated in part by disturbances in monoaminergic neurotransmission and decreased markers of synapse formation. Further exploration is required to disclose the specific mechanisms by which vitC deficiency affects memory functions in the brain.

## Figures and Tables

**Table 1:** High performance liquid chromatography (HPLC) detection of monoaminergic neurotransmitters in the hippocampus.

Group/Neurotransmitter	CTRL (*n* = 15)	DEF (*n* = 12)	*p*-Value
MHPG	0.32 ± 0.09	0.35 ± 0.10	NS
Norepinephrine	2.81 ± 1.07	2.32 ± 0.75	NS
MHPG/Norepinephrine *	0.12 (0.10; 0.14)	0.15 (0.12; 0.19)	NS
5-HIAA	0.83 ± 0.16	0.99 ± 0.27	NS
5-HT *	2.07 (1.79; 2.40)	2.2 (1.75; 2.93)	NS
5-HIAA/5-HT	0.47 ± 0.12	0.36 ± 0.08	*p* = 0.0093
HVA	ND	ND	ND
DOPAC	ND	ND	ND
Dopamine	ND	ND	ND

There is a significant decrease in the 5-HIAA/5-HT ratio in the DEF animals. Statistical analysis was performed by Student’s *t*-test. Data is displayed as mean ± SD or mean (95% confidence interval), * log-transformed data. NS: Not significant; ND: Not detectable. MHPG: 3-methoxy-4-hydroxyphenylglycol; 5-HIAA: 5-hydroxyindoleacetic acid; 5-HT: 5-hydroxytryptamine; HVA: Homovanillic acid; DOPAC: 3,4-dihydroxyphenylacetic acid; CTRL: Control animals; DEF: Deficient animals.
